# Supplementary material for: Optimization of differentiation and transcriptomic profile of THP-1 cells into macrophage by PMA
Source: PLoS One. 2023 Jul 17;18(7):e0286056. doi: 10.1371/journal.pone.0286056 (PMC10351730; doi:10.1371/journal.pone.0286056)
Supplement: S1 File — (ZIP) [file pone.0286056.s001.zip › supporting files/RNA-seq report.zip!/RNA-seq report/1_QC_result/ReadsFilter/induce-3.raw_reads.pie.pdf]

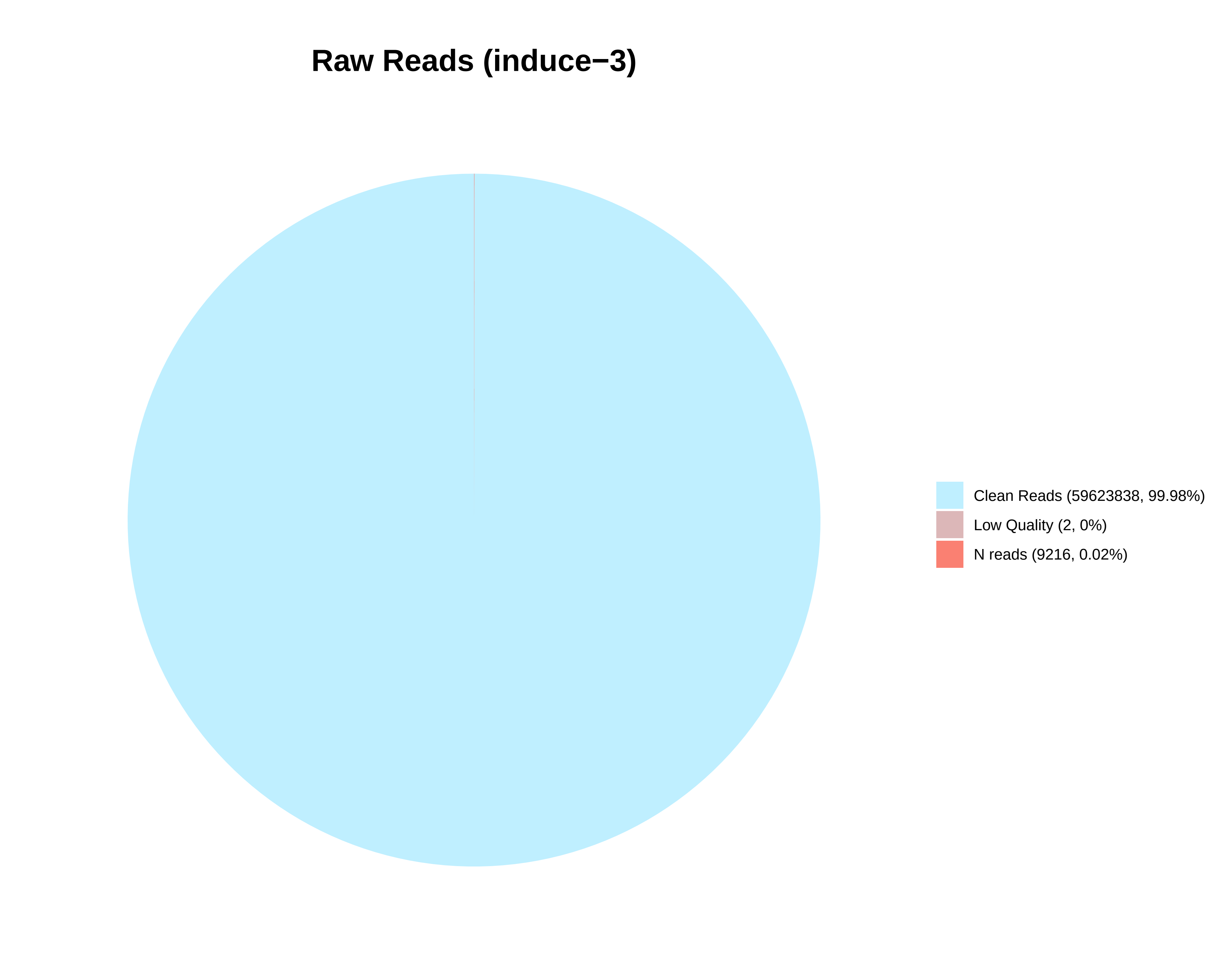
Raw Reads (induce−3)
Clean Reads (59623838, 99.98%)
Low Quality (2, 0%)
N reads (9216, 0.02%)
